# Role of Caspase-8 and Fas in Cell Death After Spinal Cord Injury

**DOI:** 10.3389/fnmol.2018.00101

**Published:** 2018-04-03

**Authors:** Daniel Sobrido-Cameán, Antón Barreiro-Iglesias

**Affiliations:** Department of Functional Biology, CIBUS, Faculty of Biology, Universidade de Santiago de Compostela, Santiago de Compostela, Spain

**Keywords:** first apoptosis signal receptor, apoptosis antigen 1, cluster of differentiation 95, tumor necrosis factor receptor superfamily member 6, caspase-8, Fas ligand, neuron, oligodendrocyte

## Abstract

Spinal cord injury (SCI) causes the death of neurons and glial cells due to the initial mechanical forces (i.e., primary injury) and through a cascade of secondary molecular events (e.g., inflammation or excitotoxicity) that exacerbate cell death. The loss of neurons and glial cells that are not replaced after the injury is one of the main causes of disability after SCI. Evidence accumulated in last decades has shown that the activation of apoptotic mechanisms is one of the factors causing the death of intrinsic spinal cord (SC) cells following SCI. Although this is not as clear for brain descending neurons, some studies have also shown that apoptosis can be activated in the brain following SCI. There are two main apoptotic pathways, the extrinsic and the intrinsic pathways. Activation of caspase-8 is an important step in the initiation of the extrinsic pathway. Studies in rodents have shown that caspase-8 is activated in SC glial cells and neurons and that the Fas receptor plays a key role in its activation following a traumatic SCI. Recent work in the lamprey model of SCI has also shown the retrograde activation of caspase-8 in brain descending neurons following SCI. Here, we review our current knowledge on the role of caspase-8 and the Fas pathway in cell death following SCI. We also provide a perspective for future work on this process, like the importance of studying the possible contribution of Fas/caspase-8 signaling in the degeneration of brain neurons after SCI in mammals.

## Introduction

Spinal cord injury (SCI) can cause permanent disability due to the dysfunction of motor, autonomic and sensory systems. There are also high economical costs associated with the care of SCI patients. In the USA, the lifetime cost of a SCI patient is between 1.1 and 4.6 million US dollars (National Spinal Cord Injury Statistical Center, 2016). So, it is of crucial importance to develop new and effective treatments for SCI patients. Nowadays, only a few treatments have been translated to the clinic: a treatment with methylprednisolone (which is still controversial), hypertensive therapy and early decompressive surgery (for a recent review see Ulndreaj et al., 2017). These treatments aim to stop further degeneration after SCI, but they only lead to limited improvements. One of the main causes of permanent deficits after SCI is due to the loss of cells (oligodendrocytes and neurons) that are not effectively replaced after the injury. Regeneration strategies are difficult to implement due to the complexity of the central nervous system; therefore, the development of neuroprotective therapies is one of the most promising strategies for clinical translation.

SCI has been divided in two stages, the primary and secondary injuries. The primary injury is caused by the mechanical forces of the traumatic event. Following the primary injury, a molecular cascade of secondary events is initiated, which expands the damage even to tissue that was not directly affected by the primary injury. Secondary injury events star within seconds of the occurrence of the primary injury and delay and progress over time. Inflammatory cells enter the injury site due to the disruption of the blood-spinal cord (SC) barrier and trigger the release of cytokines and reactive oxygen species (reviewed by Ahuja et al., 2017). Excitatory amino acids like glutamate are also massively released (Fernández-López et al., 2014, 2016) leading to elevated intracellular calcium levels. These processes cause the loss of cells by necrotic and apoptotic mechanisms. The final outcome of a SCI will depend on the extent of secondary damage; therefore, understanding the molecular pathways that lead to its progression will benefit the development of neuroprotective therapies for SCI patients.

Apoptosis is a process that occurs during development or aging and as a homeostatic mechanism to maintain cell populations in different tissues, but it is also activated after tissue damage. Cell death during secondary injury after SCI is caused in part by the activation of apoptotic mechanisms (Crowe et al., 1997; Shuman et al., 1997; Emery et al., 1998). There are two main apoptotic pathways: the extrinsic or death receptor pathway and the intrinsic or mitochondrial pathway. The extrinsic pathway involves the activation of death receptors, which leads to the activation of initiator caspases like caspase-8 or caspase-10. Several studies have shown the activation of caspase-8 in intrinsic SC cells following SCI in rodents (Casha et al., 2001, 2005; Keane et al., 2001; Takagi et al., 2003; Cantarella et al., 2010; Chen et al., 2011). More recently, work in lampreys has also shown that caspase-8 is retrogradely activated in identifiable descending brain neurons after SCI (Barreiro-Iglesias and Shifman, 2012, 2015; Barreiro-Iglesias et al., 2017). Here, we review our current knowledge on the role of caspase-8 in cell death after SCI. Since the activation of Fas receptors (also known as CD95 or APO-1) plays an important role in this process, we also focused our review on the role of this signaling pathway in caspase-8 activation following SCI. Finally, we propose new lines of work to advance our knowledge on the role of caspase-8 and Fas in cell death after SCI.

## Activation of the Fas/Caspase-8 Apoptotic Pathway in the Spinal Cord

**Figure 1:**
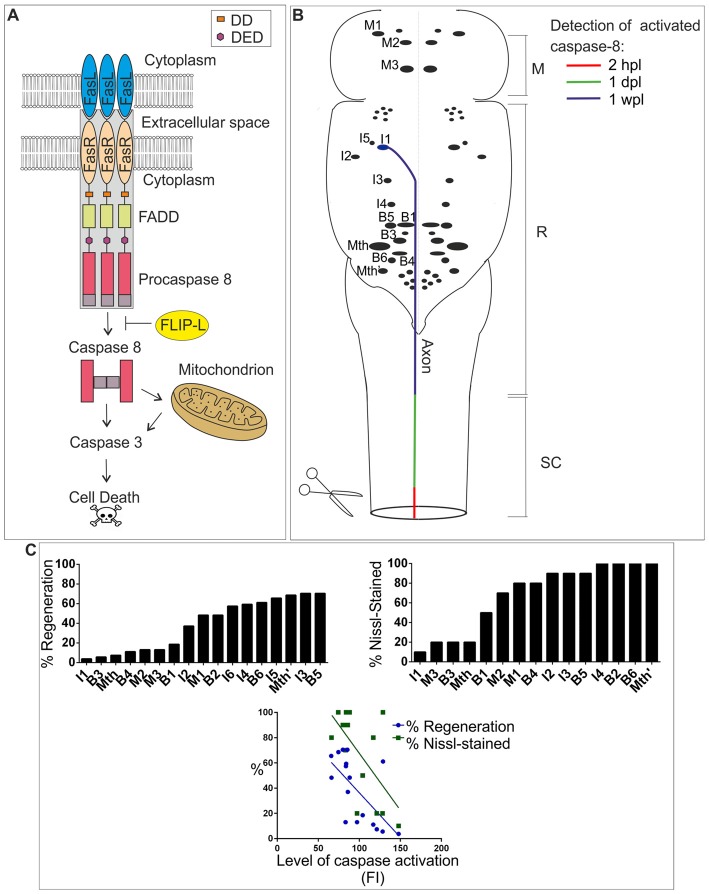
**(A)** FasL/Fas/caspase-8 signaling pathway. Binding of FasL (expressed in neurons, glial cells or immune system cells) to Fas (expressed in neurons or glial cells) induces oligomerization of the receptor, which causes the activation of the internal domain of Fas triggering FADD binding. The adaptor protein FADD binds to Fas via homophilic DD interactions. FADD recruits procaspase-8, which binds to FADD through the DED, causing the formation of death-inducing signaling complex (DISC; gray box). The formation of DISC is followed by cleavage of procaspase-8 into large and small subunits. Two large and two small subunits associate with each other to form an active caspase-8 heterodimer. The resulting mature caspase-8 is released to the cytosol and initiates downstream apoptosis directly by activating caspase-3 or indirectly through the mitochondrial pathway. FLIP-L can inhibit the activation of procaspase-8. **(B)** Schematic drawing of a dorsal view of the sea lamprey brainstem showing the location of identifiable descending neurons (for most neurons only the soma is represented). The I1 neuron is used as an example to show the progressive detection of activated caspase-8 after a complete spinal cord injury (SCI; from Barreiro-Iglesias and Shifman, 2015). Activated caspase-8 is detected first in the injured axon at the site of injury (within 2 h post-lesion, hpl), then in the axon at rostral SC levels (within 1 day post-lesion, dpl) and finally in the soma of descending neurons (1 week post-lesion, wpl). This timing of caspase-8 activation has been color-coded in the I1 neuron/axon. Rostral is to the top and the SCI site to the bottom. Abbreviations: M, Mesencephalon; R, Rhombencephalon; SC, Spinal cord. **(C)** The top graphs show the regenerative and survival abilities of identifiable descending neurons of lampreys. The regenerative ability is expressed as the percentage of times a given neuron regenerates its axon 5 mm below the site of injury 10 weeks after a complete SCI (from Jacobs et al., 1997). The survival ability is expressed as the percentage of times that a given neuron shows Nissl staining 1 year after a complete SCI (from Shifman et al., 2008). The bottom graph shows a significant correlation between the level of activated caspases (fluorescence intensity, FI) 2 wpl (from Barreiro-Iglesias et al., 2017) and the regenerative (from Jacobs et al., 1997) and survival abilities (from Shifman et al., 2008) of identifiable descending neurons. *P*-values of Pearson correlation are 0.0044 (Barreiro-Iglesias et al., 2017) and 0.0122, respectively.

Procaspase-8 is an initiator caspase that can process itself after ligation with the Fas-tumor necrosis factor family of death receptors (Kischkel et al., 1995). After biding of the Fas-ligand [FasL (or CD95L)], the Fas receptor (a 45 kDa membrane receptor) forms a death-inducing signaling complex (DISC) with the adaptor protein FADD (a member of the death domain superfamily) and procaspase-8. Then, activated caspase-8 can initiate downstream cleavage of caspase-3, among other targets, by direct or mitochondrial-dependent mechanisms (see Figure 1A). Some studies have also shown that caspase-8 activation after SCI can be mediated through other members of the TNF receptor superfamily (Cantarella et al., 2010; Chen et al., 2011), but we have focused our review on the role of FasL/Fas in caspase-8 activation after SCI.

One of the first reports showing that the Fas/caspase-8 pathway is activated after SC damage came from a study using a model of ischemic SCI (Matsushita et al., 2000). In this study, the authors developed a model of SC ischemia in mice by clamping the left subclavian artery. After ischemia, the number of Fas-positive neurons and the intensity of Fas-immunoreactivity increased. Also, ischemia induced the formation of a complex between Fas and procaspase-8 in the SC suggesting that ischemia induces the formation of DISC. Ischemia also induced and increase in procaspase-8 expression and caspase-8 cleavage/activation. Specifically, activated caspase-8 was detected in neurons (Matsushita et al., 2000). These authors did not establish how the ischemic damage leads to the formation of DISC and caspase-8 activation in neurons. But, interestingly, after cerebral ischemia there is an upregulation of Fas and the FasL (Martin-Villalba et al., 1999), suggesting that FasL release after SC ischemia could induce the formation of DISC and caspase-8 activation in neurons. This work has important implications for traumatic SCI, because the disruption of blood-vessels after SCI can also cause secondary ischemic damage.

The first reports demonstrating caspase-8 activation in intrinsic SC cells following a traumatic SCI came in 2001 from two studies by Casha et al. (2001) and Keane et al. (2001). The study by Keane et al. (2001) showed that a contusion injury at T9-T10 in rats leads to the appearance of caspase-8 immunoreactivity 6 h after the injury in neurons of the gray matter and in cells of the white matter (possibly oligodendrocytes). Immunoblots showed that this immunoreactivity correspond to the expression of the cleaved subunit of caspase-8 (Keane et al., 2001). In the same year, Casha et al. (2001) reported the first results showing the possible involvement of Fas receptors in cell death following a cervical SCI (the most common level of human SCI). These authors showed that, in rats, a clip compression C7-T1 SCI caused cell death in the SC. Apoptotic cells were mainly oligodendrocytes located along degenerating axons. Double immunohistochemistry with Fas and TUNEL revealed the presence of Fas-positive dying glia after the injury (Casha et al., 2001). Expression of FasL was observed in astrocytes and microglia. Interestingly, the appearance of Fas expression after the injury in dying glia correlated with increased levels of activated caspase-8 as revealed by western blots. Moreover, levels of FLIP-L (caspase-8 inhibitor; Figure 1) decreased after SCI at time points in which caspase-8 activation was observed (Casha et al., 2001). Similar results were later reported in mice after a T9-T10 contusion injury (Takagi et al., 2003). These authors showed that caspase-8 enzyme activity increased in the SC of mice after SCI (Takagi et al., 2003).

These earlier results suggested that activation of Fas after SCI could lead to activation of caspase-8 and cell death. However, the first true experimental demonstration showing that the activation of Fas leads to apoptosis following SCI came in 2004 with the studies by Demjen et al. (2004) and Yoshino et al. (2004). Demjen et al. (2004) showed that an acute treatment with neutralizing antibodies against FasL (CD95L) reduced neuronal apoptosis (TUNEL), promoted regeneration of corticospinal tract fibers and improved functional recovery in mice with a dorsal transection of the SC (two-thirds of the cord were transected) at T8-T9. Yoshino et al. (2004) also showed that locomotor recovery, after a contusion SCI, was improved in Fas-deficient mutant mice and that this correlated with reduced tissue damage and apoptosis. In Fas-deficient mice, fewer TUNEL positive cells undergoing apoptosis were observed (mainly neurons, although also oligodendrocytes and astrocytes). This correlated with the presence of Fas-expressing neurons after the injury in control mice and the presence of FasL-positive cells both in Fas-deficient and control mice (Yoshino et al., 2004). These functional studies confirmed that biding of the FasL to Fas receptors causes cell death after SCI and that inhibition of this signaling pathway could be a valuable therapeutic target for SCI patients.

Similar results were then obtained by Casha et al. (2005) using a model of T5-T6 clip compression SCI in mice. These authors detected post-traumatic apoptosis (activated caspase-8 and TUNEL) in neurons and oligodendrocytes. Apoptosis was reduced in Fas^Lpr/Lpr^ mutant mice. However, in contrast to the results of Yoshino et al. (2004), the reduction in apoptosis was mainly observed in oligodendrocytes. Fas deficiency led to improved locomotor recovery after SCI, which was associated to increased axonal sparing and a significant improvement in white matter myelin (Casha et al., 2005). Whether Fas leads to caspase-8 activation and apoptotic death mainly in neurons or oligodendrocytes might depend on the type of injury (contusion/transection vs. clip compression, which causes a more severe ischemia).

Subsequent work has confirmed that neutralization of Fas signaling is beneficial for recovery after SCI (Ackery et al., 2006; Robins-Steele et al., 2012). Authors of these studies developed a treatment with soluble Fas receptors to improve recovery after SCI in rats. An immediate treatment with a soluble Fas receptor after a clip compression injury at C7-T1 in rats reduced the number of TUNEL positive cells 5 days post-injury and the expression of activated caspase-3 7 days post-injury (Ackery et al., 2006). This correlated with enhanced survival of neurons and oligodendrocytes, increased axonal integrity and improved behavioral recovery (Ackery et al., 2006). Results from this work were then confirmed by Robins-Steele et al. (2012). These authors showed that a delayed treatment (which is more clinically relevant) with a soluble Fas receptor 8–24 h after a clip compression injury at C7-T1 in rats enhances oligodendrocyte and neuronal survival, reduces cavity size and improves behavioral recovery (Robins-Steele et al., 2012).

A recent study has also shown that the transgenic overexpression of p45 (another member of the death domain superfamily) increases neuronal survival, decreases retraction of corticospinal tract fibers and improves functional recovery after a transection SCI at T9 in mice (Sung et al., 2013). p45 is able to form a complex with FADD attenuating FasL-induced caspase-8 activation and cell death caused by SCI (Sung et al., 2013).

The studies in rodent models have important implications for human SCI, because the Fas pathway is also activated in primates, including humans, after SCI (Jia et al., 2011; Yu and Fehlings, 2011). In rhesus monkeys, a T11 SC hemisection induced an increase in Fas and FasL immunoreactivity in the ventral horn at time points in which the number of apoptotic TUNEL positive cells also increased (Jia et al., 2011). A large number of Fas and FasL immunoreactive neurons and glial cells also accumulated at the injury epicenter in SCs from acutely injured human patients, while these were rarely observed in control or chronically injured SCs (Yu and Fehlings, 2011). This correlated with the appearance of TUNEL and active caspase positive cells in the SC of acutely injured patients. Moreover, double immunolabeling revealed the presence of Fas or FasL and caspase-3 positive cells and of Fas or FasL expressing macrophages/neutrophils (Yu and Fehlings, 2011). These studies highlight the importance of understanding apoptotic processes to develop effective therapies for patients with SCI. As shown in this section, neutralization of FasL/Fas signaling (antibodies or soluble receptors) leads to improvements in behavioral recovery after SCI in rodent models (Demjen et al., 2004; Ackery et al., 2006; Robins-Steele et al., 2012; Table 1). In addition, several potential treatments that have been effective in animal models reduced FasL/Fas signaling and/or caspase-8 activation (Table 1), indicating that this can be a key target when developing neuroprotective therapies for SCI patients. This includes therapies that have been translated to the clinic or that are in clinical trials for SCI patients (Table 1).

**Table 1:** Table showing treatments (genetic or pharmacological) used by different authors as potential therapies for SCI and that have been shown to reduce FasL/Fas signaling and/or caspase-8 activation in animal models.

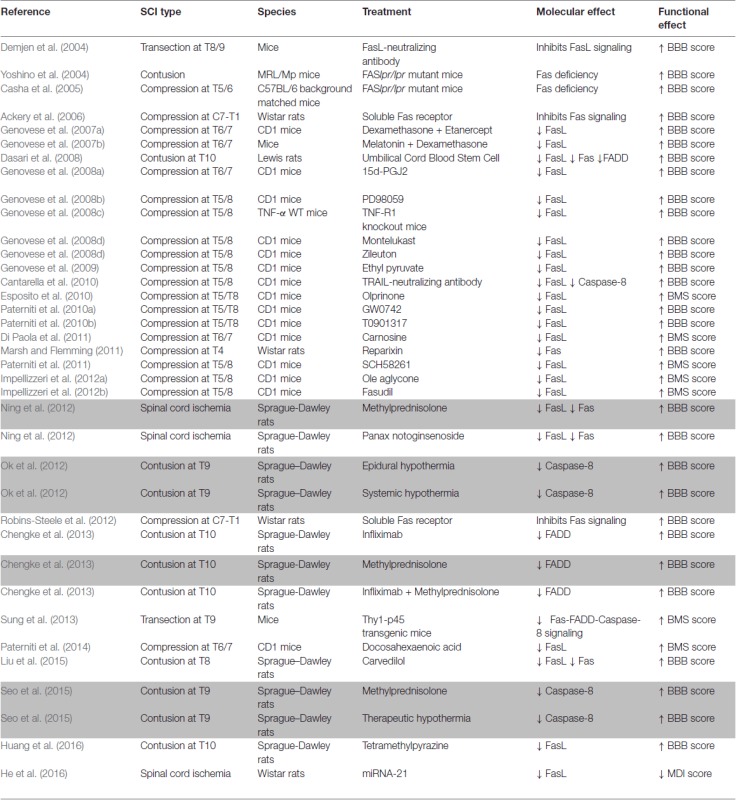

*In the “Molecular effect” column the arrows indicate a decreased expression of the corresponding molecule/s. The type of injury and the behavioral tests used to reveal behavioral improvements are also indicated in the table. Treatments that have been translated to the clinic or that are in clinical trials are highlighted in gray. Abbreviations: BBB, Basso, Beattie, Bresnahan score; BMS, Basso mouse scale score; MDI, motor deficit index score*.

## Activation of Caspase-8 in the Brain

As shown in the previous section, most studies focused on the role of caspase-8 and Fas in apoptosis of intrinsic SC cells. But, some of these studies also showed that the manipulation of FasL/Fas signaling leads to a significant increase in the regeneration/preservation of descending axons (Demjen et al., 2004; Casha et al., 2005; Ackery et al., 2006; Sung et al., 2013). These results suggest that inhibition of Fas signaling could be beneficial to preserve brain descending neurons and innervation after SCI. However, even with this evidence, no study in mammalian models has yet looked at the expression of Fas receptors or activated caspase-8 in descending neurons of the brain after SCI. We should take into account that there is still controversy on the topic of cell death in the brain following SCI. Several studies have shown the death of brain neurons after SCI in mammals, including humans (Holmes and May, 1909; Feringa and Vahlsing, 1985; Fry et al., 2003; Hains et al., 2003; Wu et al., 2003; Lee et al., 2004; Klapka et al., 2005). However, two recent studies in rats did not find any evidence of the death of corticospinal neurons after SCI (Nielson et al., 2010, 2011). The study by Nielson et al. (2011) suggested that corticospinal neurons suffer atrophy after SCI but do not die. The death/atrophy of descending neurons appears to involve and apoptotic mechanism as revealed by the appearance of TUNEL staining (although TUNEL can also label necrotic cells in some instances) and activated caspase-3 immunoreactivity in descending neurons of the brain (Hains et al., 2003; Wu et al., 2003; Lee et al., 2004).

In contrast to mammals, lampreys show an amazing capacity for functional recovery following SCI. The regeneration of descending neurons is a key event in the recovery of swimming after SCI in lampreys (see Shifman et al., 2007; Rodicio and Barreiro-Iglesias, 2012). Regenerated descending axons of lampreys are able to establish new synapses with their target neurons below the site of injury and the recovery of function depends, among other events, on the re-establishment of these connections (see Shifman et al., 2007). However, even in lampreys not all descending neurons are able to regenerate their axon after a complete SCI. The lamprey brainstem contains several individually identifiable descending neurons (Figure 1B) that vary greatly in their regenerative abilities after SCI (Figure 1C; Davis and McClellan, 1994; Jacobs et al., 1997; Barreiro-Iglesias et al., 2014). Some of these neurons are classified as “good regenerators” (i.e., they regenerate their axon more than 55% of the times) and others are considered “bad regenerators” (i.e., they regenerate their axon less than 30% of the times; Figure 1C; Jacobs et al., 1997). In recent years, mounting evidence has confirmed that descending neurons of lampreys known to be “bad regenerators” suffer a process of delayed death and are also “poor survivors” after a complete SCI (Figure 1C; Shifman et al., 2008; Barreiro-Iglesias and Shifman, 2012, 2015; Busch and Morgan, 2012; Hu et al., 2013, 2017; Zhang et al., 2014; Barreiro-Iglesias, 2015; Fogerson et al., 2016; Barreiro-Iglesias et al., 2017). The occurrence of cell death in a subset of identifiable descending neurons after SCI in lampreys was confirmed based on the disappearance of Nissl staining (Figure 1C), the loss of neurofilament expression, the absence of labeling when using retrograde tracers (Shifman et al., 2008), and the early staining of these neurons with Fluoro-Jade C (Busch and Morgan, 2012; Barreiro-Iglesias et al., 2017), which is a marker for degenerating neurons. In addition, the appearance of TUNEL staining (Shifman et al., 2008; Hu et al., 2013) and activated caspases (Figure 1C; Barreiro-Iglesias and Shifman, 2012, 2015; Hu et al., 2013; Barreiro-Iglesias et al., 2017) in the soma of axotomized descending neurons suggests that their death after SCI is apoptotic. The detection of activated caspase-8 in the first 2 weeks after the injury (Figure 1B; Barreiro-Iglesias and Shifman, 2012; Barreiro-Iglesias et al., 2017) and the lack of cytochrome-c release from mitochondria (Barreiro-Iglesias et al., 2017) indicates that the extrinsic apoptotic pathway is activated in descending neurons of lampreys after SCI. Caspase-8 activation in the soma of descending neurons of lampreys is preceded by the activation of caspases in the axotomized axons at the lesion site within the first hours after the injury (Figure 1A; Barreiro-Iglesias and Shifman, 2015; Barreiro-Iglesias et al., 2017). Caspase activation is progressively detected in descending axons at higher spinal levels and finally in the soma of descending neurons after SCI (Figure 1A; Barreiro-Iglesias and Shifman, 2015; Barreiro-Iglesias et al., 2017), which indicates that the degenerative process is initiated in the damaged axon in the SC. Taxol, which improves recovery from SCI in mammalian models (Hellal et al., 2011), prevented the appearance of activated caspase-8 in the soma of descending neurons (Barreiro-Iglesias et al., 2017) of lampreys. This indicates that death signals (or caspase-8 itself) are retrogradely transported by microtubules to the soma of descending neurons after SCI in lampreys.

The work in lampreys suggests that activated caspase-8 could also play a role in the death of descending neurons of mammals after SCI. Interestingly, in olfactory neurons of mice, appearance of activated caspase-8 in the cell body after olfactory bulbectomy depends on microtubule-based retrograde transport of activated caspase-8 by its association with dynactin p150^Glued^ (Carson et al., 2005), which might reveal conserved mechanisms with descending neurons of lampreys. In addition, the preservation of axonal projections in the SC after experimental inhibition of Fas signaling indicates that Fas receptors could play a role in the activation of caspase-8 in descending neurons after SCI.

## Conclusion

Several reports have confirmed that the activation of caspase-8 and the extrinsic apoptotic pathway is one of the mechanisms causing cell death after SCI and that the FasL/Fas signaling pathway plays a key role in this process. Surprisingly, no study has yet attempted to directly inhibit caspase-8 after SCI. This experimental approach could be of interest, especially because caspase-8 activation is not only caused by Fas receptor activation, which could lead to further improvements in recovery from SCI. Also, another aim for future work, and based on the lamprey results, should be to investigate the possible activation of caspase-8 in descending neurons of mammals after SCI and the implication of Fas signaling and microtubule retrograde transport in this process. Finally, translation of all this knowledge to pre-clinical studies or even clinical trials is of obvious and crucial importance.

## Author Contributions

AB-I wrote the manuscript with help from DS-C. DS-C prepared the figure and the table. All the work was supervised by AB-I.

## Conflict of Interest Statement

The authors declare that the research was conducted in the absence of any commercial or financial relationships that could be construed as a potential conflict of interest.
